# Gametogenesis in Malaria Parasites Is Mediated by the cGMP-Dependent Protein Kinase

**DOI:** 10.1371/journal.pbio.0060139

**Published:** 2008-06-03

**Authors:** Louisa McRobert, Cathy J Taylor, Wensheng Deng, Quinton L Fivelman, Ross M Cummings, Spencer D Polley, Oliver Billker, David A Baker

**Affiliations:** 1 Department of Infectious and Tropical Diseases, London School of Hygiene & Tropical Medicine, London, United Kingdom; 2 Wellcome Trust Sanger Institute, Hinxton, Cambridge, United Kingdom; University of Vermont, United States of America

## Abstract

Malaria parasite transmission requires differentiation of male and female gametocytes into gametes within a mosquito following a blood meal. A mosquito-derived molecule, xanthurenic acid (XA), can trigger gametogenesis, but the signalling events controlling this process in the human malaria parasite Plasmodium falciparum remain unknown. A role for cGMP was revealed by our observation that zaprinast (an inhibitor of phosphodiesterases that hydrolyse cGMP) stimulates gametogenesis in the absence of XA. Using cGMP-dependent protein kinase (PKG) inhibitors in conjunction with transgenic parasites expressing an inhibitor-insensitive mutant PKG enzyme, we demonstrate that PKG is essential for XA- and zaprinast-induced gametogenesis. Furthermore, we show that intracellular calcium (Ca^2+^) is required for differentiation and acts downstream of or in parallel with PKG activation. This work defines a key role for PKG in gametogenesis, elucidates the hierarchy of signalling events governing this process in P. falciparum, and demonstrates the feasibility of selective inhibition of a crucial regulator of the malaria parasite life cycle.

## Author Summary

The protozoan parasite Plasmodium falciparum, which causes malaria in humans, is responsible for over 1 million deaths each year. Its life cycle is complex; the asexually replicating forms, which cause disease symptoms, are quite distinct from the sexual forms, which mediate transmission between individuals via the bite of a mosquito. After a period of growth in the human host, these sexual forms (gametocytes) lie dormant until taken up by a mosquito. The change in environment from human to mosquito triggers differentiation into mature gametes. In this study, we have identified a protein kinase from the parasite that is instrumental in mediating this essential differentiation step. We have also gained insight into how this protein kinase might interact with calcium to coordinate these events. By using genetically modified malaria parasites in combination with specific inhibitors of the protein kinase, we have illustrated the feasibility of blocking development of the sexual stage of the parasite's life cycle. Development of a drug that targets this parasite stage, for use in combination with a curative drug, would be an important tool for controlling the spread of drug resistance.

## Abstract 3

We show that differentiation of malaria parasites in response to environmental signals encountered upon entering the mosquito following a blood meal is mediated by the parasite cGMP-dependent protein kinase.

## Introduction

Plasmodium falciparum is the causative agent of the most lethal form of malaria, thought to kill over a million people each year. Malaria pathology is caused by proliferation of asexual blood stage parasites, whereas transmission is mediated by an obligatory sexual life cycle phase. Gamete precursors (gametocytes) develop from asexual blood stage parasites and are thought to sequester by binding to endothelium cells. After 8–10 d, they reenter the circulation and must be taken up by a female *Anopheles* mosquito during a blood meal to continue the life cycle. Prior to activation, mature P. falciparum gametocytes are crescent-shaped, but differentiate to become spherical (known as “rounding up”) upon entering the insect midgut. Gametogenesis is stimulated in vitro by a temperature decrease, coupled with either a rise in pH [1,2] or the presence of a mosquito-derived factor [3], xanthurenic acid (XA) [4,5]. Both sexes must emerge from host erythrocytes prior to fertilisation. It is crucial to the success of transmission that gametocytes remain inactivated in the human host, yet respond immediately once inside the mosquito midgut. Upon activation, male gametocytes undergo a series of spectacular changes, including three rounds of genome replication and mitotic division, resulting in the release of eight highly motile, flagellated gametes within only 10 min [6,7]. Observation of this process (known as exflagellation) by Laveran in 1880 was one of the first major clues that malaria was caused by a parasitic protozoan [8]. In the rodent malaria parasite *Plasmodium berghei,* XA triggers an intracellular rise in Ca^2+^ concentration, which is required for gametogenesis. A Ca^2+^-dependent protein kinase (CDPK4) is known to mediate some of the effects of XA in P. berghei male gametocytes, where it is essential for the initiation of DNA replication [9]. Evidence that other second messengers may be involved in controlling this process has been reported. The products of phosphatidylinositol hydrolysis by phospholipase C have been implicated in exflagellation [10,11], and the use of pharmacological agents has provided evidence of a role for cGMP [12]. In eukaryotes, intracellular levels of cGMP are generally balanced by the opposing action of synthetic (guanylyl cyclase [GC]) and hydrolytic (phosphodiesterase [PDE]) enzymes. The P. falciparum genome contains two genes encoding biochemically active, membrane-associated GCs that are expressed in gametocytes. Each has a C-terminal, paired catalytic domain reminiscent of mammalian G protein-dependent adenylyl cyclases and an N-terminal P-type ATPase-like domain [13]. It has been observed that addition of XA to mature P. falciparum gametocyte membrane preparations elevated GC activity [14]. There are four genes encoding putative cyclic nucleotide PDEs in the P. falciparum genome, and their expression at the mRNA level is developmentally regulated [15]. A single cGMP-dependent protein kinase (PKG) enzyme is present in the parasite, which has some structural and biochemical properties unique to apicomplexan parasites [16–18]. In P. falciparum, the gene is expressed in both the asexual and sexual blood stages of the life cycle [16,19]. PKG mediates numerous cellular processes in diverse eukaryotes, ranging from changes in behavioural patterns in bees [20] to penile smooth muscle relaxation in mammals [21]. In the present study, we aimed to investigate the role of PKG in P. falciparum sexual development. We have used a genetic approach combined with specific inhibitors to show conclusively that in P. falciparum, PKG is essential for mediating initiation of gametogenesis, and we provide evidence that the enzyme may be activated in a narrow temporal window prior to Ca^2+^ signalling.

## Results

### The cGMP-PDE Inhibitor Zaprinast Can Stimulate P. falciparum Gametogenesis in the Absence of XA

**Figure 1:**
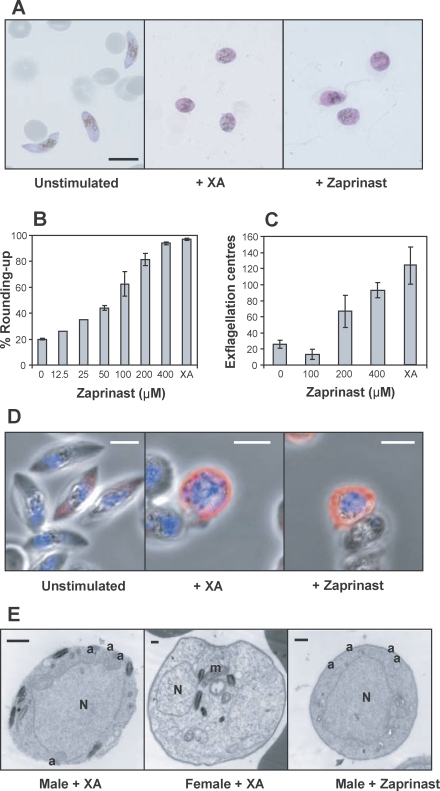
Zaprinast Can Stimulate Rounding Up and Exflagellation of Mature Gametocytes in the Absence of XA Zaprinast was added to mature gametocytes to assess the effects on gametogenesis compared to XA. The concentration of XA used was 20 μM, and the concentration of zaprinast was 400 μM, unless stated otherwise. (A) Micrographs of Giemsa-stained Stage V gametocytes prior to stimulation of gametogenesis (left panel) and after addition of XA (centre panel) or zaprinast (right panel). The scale bar indicates 10 μm. (B) Increasing concentrations of zaprinast were added to stimulate gametogenesis, and cells were scored as either round or crescent-shaped, and plotted as a percentage rounded-up. Results are based on triplicate counts of a representative experiment from the same flask of gametocytes on a single day (except for 12.5 and 25 μM, which are based on a single count only). Error bars indicate the standard error of the mean (± SEM). The experiment was carried out twice with very similar results. (C) The number of centres of exflagellation per 10,000 gametocytes was scored following addition of increasing concentrations of zaprinast. Results are based on triplicate counts of a representative experiment from the same flask of gametocytes on a single day. Error bars indicate mean ± SEM. The experiment was carried out twice with very similar results. (D) Merged confocal images of Giemsa-stained stage V gametocytes prior to stimulation of gametogenesis (left panel) and after addition of XA (centre panel) or zaprinast (right panel). Blue indicates DAPI-stained nuclei and red the anti-α tubulin antibody (Tat1) staining of cells. The scale bars indicate 5 μm. (E) Transmission electron micrographs of male or female gametocytes after stimulation of gametogenesis with XA (left and centre panels) or zaprinast (right panel). a, axonemes; m, mitochondrion; N, nucleus. Scale bars indicate 0.5 μm.

As an initial step in evaluating the potential role of cGMP in P. falciparum sexual stage development, we tested a number of inhibitors on PDE activity in parasite particulate fractions. We found that widely used PDE inhibitors, such as caffeine, IBMX, and theophylline, had little or no effect when tested on native PDE enzyme activity (see Table S1). In contrast, zaprinast, a specific cGMP-PDE inhibitor reported to interfere with asexual parasite growth [22], was an effective inhibitor (a 50% inhibitory concentration [IC_50_] of 33.7 ± 1.3 μM for gametocytes and 3.0 ± 1.2 μM for schizonts; Table S1). Importantly, when tested on live gametocytes, zaprinast stimulated rounding up in the absence of XA (Figure 1A). Zaprinast triggered both rounding up and exflagellation in a dose-dependent manner (Figure 1B and 1C), suggesting that increased intracellular cGMP levels play a role in this process. Nuclear enlargement and flagella formation, both characteristic of activated male gametocytes, were visualised by immunofluorescence analysis (IFA) (Figure 1D) and transmission electron microscopy (tEM) (Figure 1E) after stimulation with either XA or zaprinast. IFA with an anti–α-tubulin monoclonal antibody (Figure 1D) revealed a characteristic pattern of strong peripheral labelling of assembled axonemes, and transverse sections of the axonemes were also visible in electron micrographs (Figure 1E). Only a subpopulation of gametocytes was labelled by the anti–α-tubulin antibody, which may reflect a higher concentration of tubulin in male gametocytes. Taken together, these observations imply that in P. falciparum, zaprinast-induced increases in cytosolic cGMP levels can trigger gametogenesis and its constituent events.

### The Membrane-Permeable Ca^2+^ Chelator BAPTA-AM Inhibits Exflagellation, but Not Rounding Up, of P. falciparum Gametocytes

**Figure 2:**
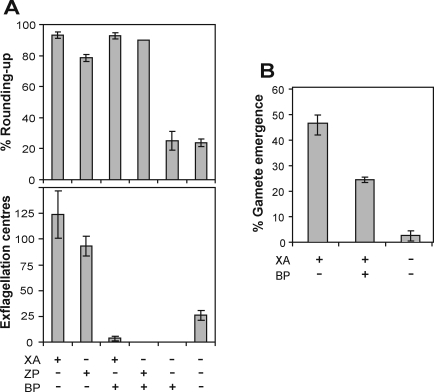
Intracellular Ca^2+^ Is Not Required for the Initial Step of P. falciparum Gametogenesis To investigate the role of intracellular Ca^2+^ in rounding up and exflagellation, stage V *Pfrh3^−^* control gametocytes were pretreated with the BAPTA-AM (BP) before stimulation of gametogenesis with either XA or zaprinast (ZP). (A) (Upper panel) Gametocytes were scored as either round or crescent-shaped, and plotted as percentage rounded-up. Results show the means of duplicate counts, and error bars indicate mean ± SEM. (Lower panel) The number of centres of exflagellation per 10,000 gametocytes was scored. Results show the means of duplicate counts and error bars indicate mean ± SEM. (B) Emergence from host erythrocytes before and after stimulation of gametogenesis with or without BAPTA-AM was scored using IFA with an anti-erythrocyte Band 3 monoclonal antibody. The proportion of cells not reacting with the antibody (indicative of complete emergence) was counted and plotted as a percentage. Results show the means from quadruplicate counts from two independent experiments, and error bars indicate mean ± SEM.

In the rodent malaria parasite P. berghei, cytosolic Ca^2+^ is mobilised within 10 s of gametocyte activation, and functions as a key second messenger for gamete egress, male cell cycle progression, and exflagellation [9]. We therefore examined the role of Ca^2+^ in P. falciparum gametogenesis activated by either XA or zaprinast. As with *P. berghei,* pretreatment of gametocytes with the membrane-permeable Ca^2+^ chelator, BAPTA-AM, blocked exflagellation (Figure 2A, lower panel) and reduced emergence of gametes from erythrocytes (Figure 2B). Gamete emergence was measured by IFA using an anti-human erythrocyte Band 3 monoclonal antibody that served as a marker for the presence of erythrocyte membrane around gametes. Zaprinast did not overcome the Ca^2+^ requirement for exflagellation (Figure 2A, lower panel), suggesting it acts upstream of Ca^2+^. Interestingly, BAPTA-AM failed to inhibit the rounding up of gametocytes following stimulation by either zaprinast or XA (Figure 2A, upper panel), indicating that this dramatic change in cellular morphology characteristic of P. falciparum gametocyte activation, might be Ca^2+^ independent and regulated through cGMP alone.

### The Anticoccidial PKG Inhibitor Compound 1 Arrests Rounding Up of Gametocytes and Exflagellation

**Figure 3:**
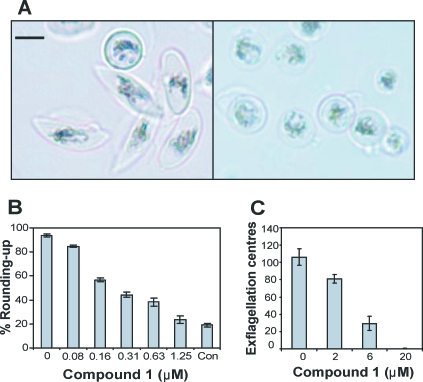
Compound 1 Inhibits Gametogenesis at Its Onset To investigate the effect of compound 1 on gametogenesis, stage V *Pfrh3^−^* gametocytes were induced with and without compound 1. (A) Light micrographs of XA-stimulated gametocytes in the presence (left panel) or absence (right panel) of 2 μM compound 1. The size bar indicates 5 μm. (B) Increasing concentrations of compound 1 were added with XA (or without XA, Con), and cells were scored as either round or crescent-shaped, and plotted as percentage rounded up. Results are based on duplicate counts from a representative experiment, and error bars indicate mean ± SEM. The results of this experiment have been reproduced on more than ten occasions. (C) The number of centres of exflagellation per 10,000 gametocytes was scored. Results are based on duplicate counts from a representative experiment, and error bars indicate mean ± SEM.

Scrutiny of the P. falciparum genome for potential mediators of cGMP, gave no obvious matches with genes encoding cyclic nucleotide-gated ion channels, but we and others have previously identified a single functional PKG (PfPKG) expressed in both asexual and sexual blood stage parasites [16,19]. We hypothesised that PfPKG could be the primary intracellular effector of cGMP in malaria parasites. We found that the *PfPKG* gene is refractory to deletion, suggesting that it is essential in the asexual blood stage parasites (L. McRobert and D. A. Baker, unpublished data). An alternative genetic approach was therefore used for functional analysis. As a first step in this strategy, we tested the effect of the trisubstituted pyrrole (4-[2-(fluorophenyl)-5-(1-methylpiperidine-4-yl)-1*H* pyrrol-3-yl] pyridine), compound 1, a highly specific ATP-competitive inhibitor of PKG in the related coccidian parasites *Eimeria* and *Toxoplasma* [18], on P. falciparum. Compound 1 inhibited the growth of asexual blood stages (IC_50_ 2.70 ± 0.17 μM; unpublished data) at a level similar to previous findings [19]. We then tested the effect of the compound on gametogenesis. Importantly, compound 1 inhibited rounding up, with gametocytes clearly retaining their distinctive crescent shape (Figure 3A, left panel). By contrast, gametocytes treated with XA alone became spherical (Figure 3A, right panel) indicating that gametogenesis had been initiated. Both rounding up and exflagellation of P. falciparum gametocytes were inhibited by compound 1 in a dose-dependent manner (Figure 3B and 3C).

### Substitution of a Key Active Site Threonine Residue Confers Compound 1 Insensitivity to Recombinant PfPKG

**Figure 4:**
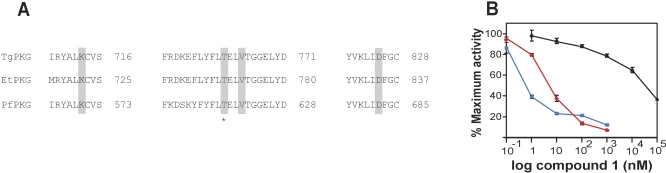
Substitution of a Key Active Site Threonine Residue Confers Compound 1 Insensitivity to Recombinant PfPKG (A) An alignment of regions of PfPKG in the ATP-binding pocket, thought to interact with compound 1, with those of other apicomplexans. Conserved active site residues highlighted in grey were predicted to approach compound 1 in a model of the Eimeria tenella PKG [29] (EtPKG). The threonine residue (T618), substituted in PfPKG in this study, is highlighted in grey and marked with an asterisk. Amino acid sequences are numbered to the right of the blocks of sequence. (B) Dose-response curves showing the effect of compound 1 on recombinant PKG enzyme activity assayed in the presence of 10 μM cGMP. Red diamonds indicate full-length WT PfPKG; black triangles, mutated PfPKG with T_618_ replaced by a glutamine residue; and blue squares, mutated PfPKG with T_618_ replaced by a methionine residue. IC_50_ values (± SEM): WT 5.8 ± 0.91 nM; T618Q mutant 17,800 ± 4,700 nM; and T618M mutant 1.5 ± 0.61 nM.

Compound 1, like most ATP-competitive inhibitors, has the potential to inhibit more than one protein kinase in the cell. To verify the specificity of compound 1, we followed a strategy used with coccidian PKGs [23] involving replacement of a key threonine residue (Figure 4A) with an amino acid with a bulky side chain to confer inhibitor insensitivity to the parasite. This substitution, critically, did not affect other aspects of biochemical activity of the coccidian enzymes and allowed unambiguous identification of the cellular events in which PKG plays a vital role. To investigate whether refractory forms of PfPKG could be engineered, we generated two alternative mutant enzymes following site-directed mutagenesis. PfPKG_T618_ was replaced with either glutamine or methionine, and expressed in Escherichia coli. Compound 1 was found to be a potent inhibitor of the wild-type (WT) recombinant enzyme (IC_50_ value was 5.79 ± 0.89 nM), whereas the mutant PfPKG harbouring the glutamine residue (PfPKG_T618Q_) was over 3,000 times less sensitive at 17.8 ± 4.7 μM (Figure 4B). This single amino acid substitution therefore confers compound 1 insensitivity to the recombinant protein. There was an unexpected (by analogy with the coccidian enzymes) decrease in IC_50_ value (1.55 ± 0.62 nM) with the mutant PKG containing M_618_ (PfPKG_T618M_) (Figure 4B), indicating that this amino acid substitution did not confer insensitivity to compound 1 in the context of the recombinant P. falciparum protein expressed in E. coli. It has been observed previously that mutation of the equivalent amino acid position (the “gatekeeper residue”) in other protein kinases can lead to unpredictable properties. Instability or even complete loss of catalytic activity has been observed, depending on the residue introduced [24]. Both mutant enzymes were therefore generated in parallel to increase the chance of obtaining a compound 1–insensitive kinase. The results indicate that only the T618Q substitution conferred a high degree of insensitivity to compound 1 and that its introduction into the parasite might facilitate identification of cellular processes in which PfPKG plays an essential role.

### Transgenic P. falciparum Gametocytes Expressing an Inhibitor-Insensitive PKG Can Round Up Normally in the Presence of Compound 1 and Compound 2 Following Stimulation by Either XA or Zaprinast

**Figure 5:**
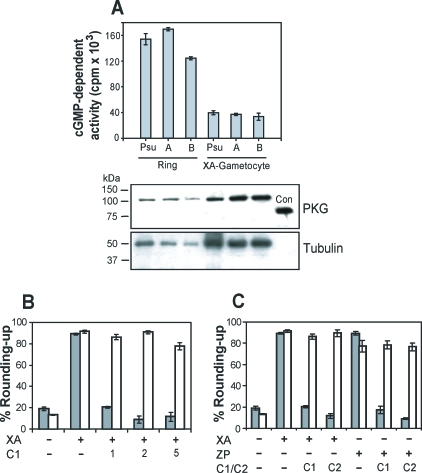
Transgenic P. falciparum Expressing an Inhibitor-Insensitive PfPKG Demonstrate an Essential Role for the Enzyme in the Initiation of Gametogenesis (A) Native cGMP-dependent kinase activity in independent clones of PfPKG_T618Q_ mutants (A and B) and the *Pfrh3^−^* (Psu) control cells were measured in both ring stage parasites and activated gametocytes in the presence of 10 μM cGMP. At least two independent experiments were carried out in triplicate, and the data displayed are from a representative experiment (error bars indicate mean ± SEM). A western blot of each sample is shown beneath, incubated with an anti-human PKGIα antibody. Lanes contain approximately 2 × 10^8^ ring stage parasites or 2 × 10^6^ activated gametocytes. One quarter of these amounts was used in native PKG assays. A recombinant, N-terminally truncated PfPKG (87 kDa) is included as a positive control (Con). Equivalent loading was assessed by reprobing the blot with the anti–α-tubulin antibody Tat-1. (B) Compound 1 (1 μM, 2 μM, and 5 μM) was added along with XA to WT (grey bars) and PfPKG_T618Q_ (clone A) gametocytes (white bars); cells were scored as either round or crescent-shaped, and plotted as percentage rounded-up. Results show data from duplicate counts from a representative experiment, and error bars indicate mean ± SEM. (C) Quantification of rounding up upon stimulation of WT (grey bars) and PfPKG_T618Q_ (clone A) gametocytes (white bars) with either XA or zaprinast (ZP) in the presence of either 2 μM compound 1 (C1) or 2 μM compound 2 (C2). Results show data from duplicate counts from a representative experiment, and error bars indicate mean ± SEM.

Compound 1–resistant P. falciparum parasites were then generated using an allelic replacement strategy. Asexual 3D7 ring stage parasites were transfected according to standard procedures using the pHH1 plasmid in conjunction with the antifolate WR99210 for drug selection [25]. Genotype analysis confirmed that a single crossover homologous recombination had occurred, introducing the desired nucleotide substitution into the *PfPKG* gene downstream of its own promoter (Figure S1). In the absence of compound 1, the mutant and control cell lines had equivalent properties in terms of their rates of asexual replication (Figure S2) and gametocyte development, confirming that the mutant PKG was fully functional. An approximately 2-fold increase in the expression level (possibly due to the presence of a heterologous 3′ untranslated region following allelic replacement) of PfPKG in activated PfPKG_T618Q_ gametocyte preparations was measured in western blots, but cGMP-dependent kinase activity levels were equivalent in mutant and control lines (Figure 5A). The difference in PKG band intensity obtained in western blots between life cycle stages probably reflects a higher level of PKG expression in gametocytes compared to rings, but the higher specific activity in rings is reproducible. However, the purpose of this figure is to provide confirmation that PKG activity levels measured in control and mutant clones were similar at a given life cycle stage.

In a key experiment, the mutant cell lines were tested for their ability to round-up in the presence of compound 1. Addition of 1–5 μM compound 1 to control gametocytes reduced rounding up to background levels. Crucially, we observed that gametocytes from PfPKG_T618Q_ clones rounded up to almost normal levels in the presence of 1–5 μM compound 1 after stimulation with XA (Figure 5B). These data confirm that expression of PfPKG_T618Q_ in mutant parasites reduces their sensitivity to compound 1. The results show clearly that PfPKG is the primary target of compound 1 during initiation of differentiation and demonstrate that this enzyme plays an essential role in stimulating gametogenesis. We obtained similar results using a second inhibitor (the imidazopyridine, 4-[7-[(dimethylamino)methyl]-2-(4-fluorophenyl)imidazo[1,2-*a*]pyridine-3-yl]pyrimidin-2-amine, compound 2 [26]) (Figure 5C), confirming an essential role for PfPKG in gametogenesis. Furthermore, the absence of inhibitory effects of compound 1 and compound 2 upon stimulation of PfPKG_T618Q_ gametocytes with either zaprinast or XA (Figure 5C) indicates that the ability of both compounds to trigger gametogenesis is PKG dependent. Measurements of rounding up using a range of concentrations of compound 1 and 2 showed that PfPKG_T618Q_ gametocytes had an approximately 10–20-fold lower sensitivity to the inhibitors than control cells. IFA and tEM analysis revealed that the compound 1–treated control cells showed no evidence of DNA synthesis (enlarged male nuclei), axoneme formation, or emergence from red cells and thus resembled unstimulated gametocytes. This confirms that inhibition of PfPKG blocks gametogenesis at its onset.

Although we were able to measure cGMP-dependent kinase activity in cell lysates, we were unable to observe any differential compound 1 sensitivity in this activity between control and mutant lysates. Both had equivalent levels of sensitivity to compound 1. A likely explanation is that the cGMP-dependent activity measured in lysates includes a PKG-activated, compound 1–sensitive downstream kinase activity, with the result that the difference in compound 1 sensitivity of PKG itself between the two cell lines is masked. This is consistent with our observation that although both compound 1 and 2 inhibited exflagellation in a dose-dependent manner, there was no significant difference in sensitivity to either compound measured in control and PfPKG_T618Q_ cell lines in terms of inhibition of exflagellation levels (unpublished data). This suggests that the compounds may inhibit additional protein kinases (downstream of PKG) that are required for other constituent events of exflagellation, but not for rounding up. Candidates include members of the CDPK family [26].

A cloned line of PfPKG_T618M_ was also generated and tested for the effects of compound 1 on rounding up and exflagellation. The relatively small (<4-fold) increase in sensitivity to compound 1 that was unexpectedly observed in the recombinant enzyme was not reflected in the parasite. There was no significant difference in sensitivity to compound 1 compared to control cells, confirming that the T618M substitution did not confer insensitivity to compound 1. By contrast, the greatly decreased sensitivity to compound 1 conferred by the T618Q mutant (>3,000-fold in the recombinant protein) was reflected in a highly significant (analysed by Poisson Regression with Stata 9.2; StataCorp) decrease in inhibitor sensitivity in the parasite. Therefore, only the T618Q mutant proved to be a useful tool for functional studies. Analysis of rounding up in the presence of varying compound 1 concentration (0.1–20 μM) from replicate experiments for both mutants compared to WT is presented in Figure S3 and Table S2.

**Figure 6:**
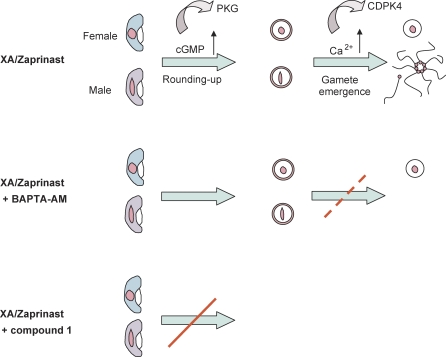
A Schematic Representing the Progression of Gametogenesis and the Proposed Hierarchy of Signalling Events That Occur P. falciparum gametogenesis can be stimulated in vitro by addition of XA or zaprinast combined with a decrease in temperature. Within 2 min, a profound morphological change occurs whereby both male and female gametocytes transform from crescent-shaped to spherical. This process can still take place upon chelation of intracellular Ca^2+^ with BAPTA-AM, but gamete emergence is reduced and exflagellation is completely inhibited. By contrast, the primary rounding-up step (and subsequent progression) is completely halted by specific inhibition of the parasite PKG. This protein kinase therefore plays an essential role in mediating the initiation of gametogenesis, and it may act upstream of or in parallel with Ca^2+^ signalling. The involvement of CDPK4 in this process has been demonstrated for P. berghei.

The combined results of this study indicate that PfPKG is essential for the Ca^2+^-independent differentiation of P. falciparum gametocytes from crescent-shaped to spherical, whereas Ca^2+^ is required for other constituent events of gametogenesis including exflagellation (Figure 6).

## Discussion

As *Plasmodium* gametocytes transit from the mammalian blood stream to the mosquito midgut, they encounter a radically distinct environment. Gametogenesis is triggered rapidly in response to signals in the new environment so that fertilisation can proceed. The molecular details, however, of how reduced temperature might combine with XA or pH changes to bring about differentiation are largely unknown. In this study, we have demonstrated a role for cGMP and PKG in the initiation of gametogenesis by XA in P. falciparum. We have further concluded that although Ca^2+^ is also essential for this process, it is possible that activation of PKG may precede Ca^2+^ in the hierarchy of signalling events.

### A Role for cGMP in Regulating Differentiation

We found that PDE inhibitors previously reported to promote small increases in exflagellation in *Plasmodium* [12,27] did not inhibit P. falciparum native PDE activity, whereas zaprinast (a specific cGMP-PDE inhibitor) was effective in the mid micromolar range. Zaprinast (400 μM) induced both rounding up of gametocytes and exflagellation at levels almost equivalent to those triggered by XA, suggesting that increases in endogenous cGMP levels could indeed stimulate exflagellation as previously reported [12]. Zaprinast has previously been demonstrated to effectively inhibit PDEs from Trypanosoma brucei [28] and a P. falciparum PDE expressed during the asexual life cycle stages [22]. The insensitivity of the protozoan PDEs to several potent inhibitors of mammalian PDEs probably reflects subtle differences in the enzyme active sites.

Our results show that the underlying cellular events that accompany XA-induced rounding up also take place upon addition of zaprinast and thus confirm the specificity of the morphological change. Furthermore, the validity of the rounding up stimulated by zaprinast was verified unequivocally by the fact the male gametocytes were able to exflagellate. The specificity of zaprinast on the cGMP pathway was confirmed when used in combination with mutant cell lines expressing a PKG allele conferring reduced sensitivity to inhibitors (see below).

### PKG Is Essential for the Initiation of Gametogenesis in P. falciparum

Since the parasite PKG proved refractory to deletion, we generated an inhibitor-insensitive mutant line of P. falciparum by allelic replacement for functional studies. A similar approach was used previously to determine the role of PKG in related coccidian parasites [23]. The strategy relies on a key threonine residue found in apicomplexan PKGs (but few other protein kinases) that forms the basis of selectivity of the fluorophenyl group of compounds 1 and 2. Replacement of the threonine with an alternative residue containing a bulky side chain is thought to prevent access of the inhibitors to a binding pocket that overlaps the ATP-binding site and thereby confers insensitivity to the compound [26,29]. A key point is that in the absence of inhibitors, the PfPKG_Q618_ mutant clones behaved normally in terms of asexual replication and sexual development. Levels of rounding up and exflagellation were also indistinguishable from WT parasites, suggesting that the mutant PKG is fully functional. This also confirms that there have been no adverse effects due to the genetic modification itself. Upon addition of compounds 1 and 2, dramatic differences between control parasites and mutants were noted in terms of their ability to round up following stimulation by either XA or zaprinast. We conclude that PKG is the primary target of the compounds and that PKG activity is essential for the initiation of gametogenesis. This also demonstrates that the effects of XA and zaprinast are PKG dependent and supports the view that XA, cGMP, and PKG are all components of the same gametocyte activation pathway in P. falciparum. Differences between control and mutant parasite lines in their sensitivity to compounds 1 and 2 during exflagellation, however, were not pronounced. This suggests that other protein kinases, sensitive to compounds 1 and 2, may be functional as exflagellation proceeds. A potential candidate is the P. falciparum ortholog of PbCDPK4, the Ca^2+^-dependent protein kinase essential for exflagellation in P. berghei [9]. In support of this hypothesis, it is of note that the CDPK4 ortholog from *Toxoplasma* and *Eimeria* (designated CDPK1) is a secondary target of compound 2 [26]. PfCDPK4 has a serine residue at the key position that is potentially compatible with inhibitor sensitivity.

### Intracellular Ca^2+^ Is Required for Exflagellation, but Not Rounding Up, of P. falciparum Gametocytes

We used the membrane-permeable Ca^2+^ chelator BAPTA-AM to show that, as with P. berghei [9], intracellular Ca^2+^ is essential for exflagellation in P. falciparum. However, it is clear that rounding up, an early event during P. falciparum gametogenesis, which requires PKG, is not sensitive to inhibition by BAPTA-AM. Cellular Ca^2+^ is therefore not essential for PKG activation and thus cannot be the key upstream regulator. By contrast, this step is sensitive to specific PKG inhibitors and is therefore PKG dependent. The early events in P. falciparum gametogenesis mediated by PKG are assumed to occur in both sexes, because compound 1 prevents the change of shape in over 90% of gametocytes, therefore likely reflecting a population of both males and females. In P. berghei, it has been shown that BAPTA-AM can block all the constituent events of both male and female gametogenesis, indicating that they are Ca^2+^ dependent [9]. It is therefore unclear whether the Ca^2+^-independent nature of P. falciparum rounding up represents a species-specific difference or whether it reflects the fact that rounding up in P. berghei is not such a morphologically distinguishable event since the crescent shape, characteristic of P. falciparum gametocytes, is lacking in rodent malarias.

Our observation that in P. falciparum constituent events of gametogenesis, that are Ca^2+^ dependent, can be triggered effectively by the PDE inhibitor zaprinast alone, would be consistent with a hierarchical second messenger cascade in which the cGMP effector PKG functions as a master switch during gametocyte activation that acts upstream of Ca^2+^ mobilisation. However, alternative models in which Ca^2+^ and cGMP have parallel functions cannot currently be ruled out. Interestingly, crosstalk between both second messenger pathways has been suggested to regulate other aspects of apicomplexan biology, gliding motility, and invasion in Toxoplasma gondii.

All constituent events of gametocyte activation in P. falciparum and P. berghei require gametocytes to be exposed to two coinciding stimuli, one of which must be a drop in temperature, the other can be XA or a rise in extracellular pH [1–5]. The mobilisation of cellular Ca^2+^ in P. berghei occurs approximately 10 s after gametocytes have been exposed to activating conditions [9]. A cGMP-dependent signalling pathway operating upstream of calcium mobilisation would thus have to become active within 10 s. It may either serve to integrate the different activating stimuli or may only be initiated once these have been integrated by unknown receptor mechanisms. How, at the molecular level, Ca^2+^ and cGMP-dependent signalling pathways may be linked to each other and to upstream activators remains unknown, which is true also of the GC and PDEs that control the cGMP level in gametocytes. It is clear, however, that downstream of the initial activation, different Ca^2+^- and cGMP-dependent effectors are required to drive gametocyte differentiation. Combined evidence from P. berghei and P. falciparum suggests that at least three such effector pathways exist: (1) a PKG-dependent, Ca^2+^-independent pathway that mediates rounding up of P. falciparum gametocytes; (2) a Ca^2+^-dependent pathway (which may also be PKG dependent) that initiates cell cycle progression in microgametocytes through CDPK4 [9]; and (3) a calcium-dependent pathway (which may also require PKG) that regulates CDPK4-independent constituent events, such as emergence, in gametocytes of either sex [9].

### PKG Plays Numerous Important Roles in Diverse Organisms

A role for PKG in secretion of adhesins required for gliding motility and host cell invasion in *Eimeria* sporozoites and *Toxoplasma* tachyzoites has been demonstrated [23]. Interestingly, a role for cGMP in P. berghei ookinete gliding motility has been suggested following deletion of a *GC* gene (*PbGCβ*) in P. berghei [30]. Deletion of this gene had no measurable effect on gametogenesis in the rodent malaria parasite, which is consistent with our results following disruption of *PfGCβ* in P. falciparum [31]. However, a second structurally related *GC* gene expressed in P. falciparum gametocytes [13] may provide functional redundancy. Interestingly, disruption of a P. falciparum cGMP-PDE gene (*PfPDEδ*) that is highly up-regulated in gametocytes leads to prematurely high intracellular cGMP levels and a greatly reduced ability to undergo gametogenesis [31], suggesting that regulation of cGMP levels, particularly at the level of its breakdown, is important for normal gametogenesis.

A number of physiological roles have been identified for PKG in the organisms studied to date. In the single-cell alga *Chlamydomonas*, PKG has a key role in the signalling events induced by flagellar adhesion via interaction of surface agglutinins during fertilisation [32,33]. PKG is involved in changes in patterns of foraging behaviour in insects such as *Drosophila* [34], bees [20], and ants [35], and also in the nematode Caenorhabditis elegans [36]. One of the mammalian PKG isoforms (cGKI) is found in a number of tissue types, including all smooth muscles. The association of PKG function and Ca^2+^ levels in smooth muscle tone has been the subject of intense study [37]. Mammalian PKG also plays a key role in penile smooth muscle relaxation and has been shown to phosphorylate a number of Ca^2+^-binding proteins involved in this process [21].

Our demonstration that PfPKG has a crucial role in regulating gametogenesis in P. falciparum now provides a framework for identifying the downstream proteins involved in differentiation. It will be intriguing to compare the PKG substrates that mediate the underlying events of gametogenesis in *Plasmodium* with those that mediate parasite invasion and egress in other apicomplexans in which PKG plays a role. Compounds 1 and 2 are prototype inhibitors of apicomplexan PKG which have been shown to cure chickens of *Eimeria* infection and mice of infection by T. gondii. Compound 1 also delays the onset of P. berghei infections in mice [18,19,26]. Our data predict that in addition to targeting asexual erythrocytic stages, a drug inhibiting *Plasmodium* PKG could also block parasite transmission to the mosquito, a highly desirable property that would help limit the spread of any drug-resistant parasites. Transmission-blocking drugs would be a powerful tool for reducing the malaria burden in areas endemic for P. falciparum.

## Materials and Methods

All reagents were from Sigma-Aldrich unless otherwise stated.

### Cell culture and gametogenesis assays.

P. falciparum gametocytes were produced from the 3D7 clone in human A+ erythrocytes with 10% human serum (National Blood Service), and enriched using Nycodenz (Nycoprep 1.077; Axis-Shield) centrifugation as previously described [38]. For gametogenesis, cells were resuspended at 5 × 10^6^ parasites per millilitre and induced with either 20 μM XA or 25–400 μM zaprinast in complete medium, and a reduction (of >5 °C) in temperature. For gametogenesis assays, 10 min post induction, cells were mounted on a wet smear. Rounding up was observed for a total of 200 cells, and exflagellation for a total of 10,000 cells over 10 min. Test reagents including zaprinast, BAPTA-AM, compound 1, and compound 2 were resuspended in DMSO as stock solutions and diluted in complete medium for gametogenesis assays, with the XA stock resuspended in RPMI 1640. The maximum final DMSO concentration in the gametogenesis medium was 0.4%, which was used as a negative control. Negative control cells were exposed to medium that was at exactly the same temperature as the test cells, but no rounding up or exflagellation was seen in the negative controls. Compound 1 and compound 2 were added at the point of activation with either XA or zaprinast, whereas cells were preincubated with 100 μM BAPTA-AM as previously described [9]. Statistical multivariate analysis was carried out using Poisson regression (Stata 9.2; StataCorp) to compare the effects of genetic background (mutant vs. WT) and experimental replicate (included as categorical data in the analysis) on the rates of rounding up at individual drug concentrations. For each drug concentration, the mutants were tested separately against the WT control, and the resultant output was anti-logged to give the relative risk (and 95% confidence interval) for rounding up with genetic background.

### Transfection.

Allelic replacement of PfPKG was performed using standard transfection techniques [25]. Constructs based on the pHH1 plasmid were produced as follows: The *hsp86* 5′ region was excised from pHH1 using XhoI and BglII, and replaced with the 1.7-kb 3′ fragment of *PfPKG* (amino acids [aa] 286–853) mutated at the T618 position to either methionine or glutamine [17]; see Figure S1 for further details. Asexual ring stage parasites were transfected with either pHH1-PKG-M or pHH1-PKG-Q. The presence of human *dihydrofolate reductase* selectable marker in the plasmids allowed selection of integrants with on/off drug cycling with the antifolate WR99210 (10 nM). Parasites were cloned by limiting dilution prior to genotype verification by Southern analysis and PCR. A 3D7-derived *Pfrh3* KO line, used as a control, was generated using the pHHTK plasmid as previously described [39]. This cell line had been in culture for the same length of time and under the same conditions as the mutant cell lines. It was indistinguishable from WT in terms of asexual and sexual growth and development.

### IFA and tEM analysis.

For IFA, 3 × 10^6^ mature P. falciparum gametocytes were fixed in paraformaldehyde according to a method used for P. berghei [40]. Cells were washed thoroughly and allowed to settle on wells coated with 0.01% poly-l-lysine solution. Primary antibodies, either mouse anti–α-tubulin (Tat-1; a gift from Keith Gull, University of Oxford) or mouse monoclonal anti-Band 3 (Abcam) were used at a concentration of 1:50, followed by a 1:10,000 dilution of Alexa Fluor 594 goat anti-mouse IgG (Molecular Probes). Vectashield (Vector Laboratories) containing DAPI allowed the cells to be visualised using a Zeiss Axovert LSM 510 confocal microscope. Images were captured using LSM 510 software (Carl Zeiss MicroImaging). For tEM, 10^8^ erythrocytes (at 5%–10% gametocytaemia) were fixed in 2.5% glutaraldehyde/2% paraformaldehyde [41] after induction of gametogenesis by XA or zaprinast. Pellets were treated with 1% osmium tetroxide and embedded in TAAB hard resin. Ultra-thin resin sections were stained with Reynolds lead citrate and examined on a Jeol 1200EX Mark II transmission electron microscope at 80 kV. Digital images were recorded using a side-mounted 1K 1.3M pixel High Sensitivity AMT Advantage ER CCD camera system.

### Growth inhibition assays.

Asexual parasite growth assays were carried out to obtain the IC_50_ of test compounds using inhibition of incorporation of [^3^H]-hypoxanthine [42], and to compare growth rates between transfected clones. Briefly, in 96-well plates, each test well was dosed at early trophozoite stage with 10 μl of [^3^H]-hypoxanthine (GE Healthcare) to a final concentration of 0.2 μCi. The radioactivity of the [^3^H]-hypoxanthine incorporated into parasite nucleic acid over 24 h was determined relative to the WT controls using a Wallac 1450 Microbeta scintillation β-counter (Perkin-Elmer). This assay was done in triplicate and repeated at least three times.

### PKG activity assay.

The activity of recombinant and native PKG was measured using a modification of standard procedures [18,19]. The gene encoding full-length PfPKG was cloned into the pTrcHis expression plasmid (Invitrogen). Substitution of specific amino acids of PfPKG was carried out using a QuickChange Multi Site-Directed Mutagenesis kit (Stratagene) according to the manufacturer's instructions. Expression, affinity purification of E. coli–expressed recombinant proteins, and PKG activity assays were performed as previously described [17]. All experiments were performed at least twice in triplicate. PKG measurements in native P. falciparum proteins were performed using an adaptation of a previously described assay [19]. Briefly, ring stage parasites were exposed to 0.15% saponin solution in PBS to free the parasites from their host cells. Both gametocyte and ring stage parasite pellets were lysed in HBS buffer (50 mM Hepes [pH 7.4], 1 mM EDTA, 1% Nonidet P-40, 10 mM NaF, 0.1 mM sodium orthovanadate, 1 mM dithiotreitol) supplemented with a 1/100 dilution of Sigma-Aldrich Protease Inhibitor Cocktail. Parasite lysis was confirmed by microscopic examination of Giemsa-stained thin smears. Kinase reactions were carried out in 40-μl volumes in the presence of 250 μM kemptide (LRRASLG; AnaSpec), and 0.01 μM [^32^P]-ATP (3,000 Ci/mmol; GE Healthcare). The reactions were initiated by the addition of 2 μl of lysate and incubated at 30 °C for 45 min. Reactions were terminated and stored at −80 °C. Radiolabelled peptide was detected on 96-well multiscreen phosphocellulose plates (MAPH-NOB; Millipore) by scintillation counting on a Wallac 1450 Microbeta scintillation β-counter (Perkin-Elmer). Native erythrocyte PKG activity present in the lysates was quantified and did not contribute towards the observed parasite PKG activity. In the gametocyte lysate samples, erythrocyte contamination was between 1.1% and 2.3%. The number of rings and gametocytes were 4.9 × 10^7^ and 5.3 × 10^5^,_,_ respectively. Counts were normalised by overall protein amounts in all six fractions.

### Western blotting.

Recombinant protein and parasites were prepared as described for the PKG activity assay. Protein samples were lysed in reducing sample buffer, boiled for 10 min, run on a 10% polyacrylamide gel, and transferred to nitrocellulose membrane according to standard procedures. Equal total protein concentrations of all six fractions were loaded. Membranes were blocked in 2% ECL Advance blocking powder (GE Healthcare) in Tris-buffered saline (TBS [pH 7.6]) with 0.1% Tween 20 for 1 h according to the manufacturer's instructions. Blots were probed with the anti-human PKGIα peptide antibody (C-terminal 657–671, PK10; Calbiochem) at a concentration of 1:10,000. This antibody cross-reacts with PfPKG by virtue of a common C-terminal sequence motif. The antibody did not react with uninfected human erythrocyte proteins. A HRP-conjugated goat anti-rabbit antiserum was used as the secondary antibody (BioRad) at a concentration of 1:30,000. Signals were detected using the ECL Advance chemiluminescence kit and ECL Hyperfilm (both GE Healthcare) according to the manufacturer's instructions. Bands were quantified using ImageQuant software (Molecular Dynamics).

### PDE assays.

PDE activity in native parasite fractions was measured using a modification of a previously published method [43]. Parasites were frozen in liquid nitrogen and stored at −80 °C until use. Parasites were resuspended in 500 μl of lysis buffer (20 mM hepes and 250 mM sucrose [pH 7.0]), subjected to five cycles of freeze–thaw in liquid nitrogen and pelleted at 100,000 *g* for 30 min. Particulate fractions were resuspended in lysis buffer containing EDTA-free protease inhibitors (Roche). PDE assays were carried out in triplicate wells of a 96-well plate in the presence of [^3^H]-labelled cGMP (GE Healthcare) for 30 min at 37 °C. Reactions were terminated by boiling the plate for 1 min, followed by a 3-min centrifugation at 900 *g.* One unit of alkaline phosphatase was added to each well and incubated for 30 min at 37 °C. [^3^H]-labelled guanosine was separated from the radiolabelled cGMP substrate using ion exchange (BioRad AG 1 × 8 resin). Supernatants containing the [^3^H]-labelled guanosine product were added to scintillation fluid (Optiphase Supermix; Wallac). Scintillation was measured using a Wallac 1450 Microbeta Liquid Scintillation Counter (Perkin Elmer) and PDE activity was expressed in picomoles cGMP/minute/milligram protein. Inhibition assays were carried out in the presence of compounds dissolved in DMSO. PDE assays for specific activity and IC_50_ determination were carried out at a native lysate dilution that gave 30% cGMP/cAMP hydrolysis.

## Supporting Information

Figure S1 Allelic Replacement of the *PfPKG* Gene (A) Plasmid construct pHH1-PKG-Q/M used to mediate allelic replacement. E, EcoRI; *hDHFR*, human dihydrofolate reductase selection cassette; *PbDT* 3′, translational termination sequences derived from *P. berghei DHFR/TS* 3′ untranslated region; *PKG* 3′, an N-terminally truncated fragment of the PfPKG coding region containing the T618Q or the T618M substitution (star); Xb, XbaI. Dashed line represents the plasmid backbone.
(C) Representation of the single crossover integration event that introduced the T618Q substitution into PfPKG. The black bar beneath depicts the position of the hDHFR probe used for Southern analysis (lanes 1–3 on the Southern blot). Arrows show the positions of the forward and reverse primers used to amplify then sequence across the integration event, confirming the presence of the mutated allele. The predicted restriction fragment sizes are indicated. Black flag represents the *PfPKG* promoter.
(B) The WT 3D7 *PfPKG* locus. Introns are not shown.
(D) Representation of the single crossover integration event that introduced the T618M substitution into PfPKG. The black bars beneath depict the positions of the *PfPKG* 3′ probe used for Southern analysis (lanes 4–6 on the Southern blot).
(E) Southern blot analysis of WT and PfPKG mutants. Band sizes are in kilobases.
(727 KB EPS)

Figure S2 Asexual Blood Stage Parasite Growth Is Not Impaired in Clones Expressing the PfPKG_T618Q_ Mutant Allele Asexual growth of WT and PfPKG_T618Q_ clone A and PfPKG_T618Q_ clone B was monitored via incorporation of [^3^H]-hypoxanthine and plotted as counts per minute (cpm). Data show the means of triplicate counts from three independent experiments, and error bars indicate ± the standard error of the mean (SEM). In addition to the above measurements, the relative growth of asexual parasites derived from allelic replacement and control clones was evaluated on numerous occasions (*n* > 10) prior to initiating gametocyte growth. On all occasions, growth rates between clones were very similar, allowing gametocytogenesis to be initiated simultaneously for subsequent gametogenesis assays in mutants and controls.
(256 KB EPS)

Figure S3 Mean Rounding-Up Values for Mutant Clones and WT Plotted against Compound 1 Concentration Rounding up of mutant clones PfPKG_T618M_ (21C11) and PfPKG_T618Q_ (4D2) compared to WT over a range of compound 1 concentrations (0.1–20 μM). Error bars indicate ± SEM. Data were analysed using the Prism package (GraphPad software).
(724 KB EPS)

Table S1 The Effect of PDE Inhibitors on Particulate Fractions Isolated from P. falciparum Sexual and Asexual Blood Stage Parasites IC_50_ values (the concentration of compound that gave 50% inhibition of native PDE activity) are mean ± SEM in micromolar and were calculated using the Prism software package (GraphPad Software). Percentage inhibition data were fitted to a sigmoidal dose-response curve using nonlinear regression. n.d., not determined; >200, sufficient inhibition was not observed for an IC_50_ calculation. The specific activity of cGMP hydrolysis measured in the individual life cycle stages (at a substrate concentration of 0.82 μM) is listed in the lower row. Specific activity data are mean ± SEM in picomoles cGMP/minute/milligram.
(43 KB DOC)

Table S2 Poisson Regression Analysis for Compound 1 Sensitivity of Rounding Up of Both Mutants Compared to WT Each rounding-up measurement was made by counting 200 cells and scoring for rounding up (or not rounding up). At each compound 1 concentration, rounding-up replicate counts for both clones were compared to WT values separately by Poisson regression analysis using the Stata 9.2 package (StataCorp). Where *n* < 2 (indicated by n.d.), data were excluded from the analysis. Compound 1 concentration (comp1) is shown in micromolar. The *p*-values from this analysis and the relative risk (RR) of rounding up with genetic background (and 95% confidence intervals [CI]) are shown.
(38 KB DOC)

### Accession Numbers

The GenBank (http://www.ncbi.nlm.nih.gov/Genbank) accession number for *EtPKG* is AF411961 and for *TgPKG* is AF413570.

The PlasmoDB (http://plasmodb.org/plasmo/) accession number for *PfPKG* is PF14_0346.

## Abbreviations

CDPK: calcium-dependent protein kinase
GC: guanylyl cyclase
IC_50_: 50% inhibitory concentration
IFA: immunofluorescence analysis, PDE, phosphodiesterase
PKG: cGMP-dependent protein kinase
tEM: transmission electron microscopy
WT: wild type
XA: xanthurenic acid
