# Supplementary material for: Gametogenesis in Malaria Parasites Is Mediated by the cGMP-Dependent Protein Kinase
Source: PLoS Biol. 2008 Jun 3;6(6):e139. doi: 10.1371/journal.pbio.0060139 (PMC2408617; doi:10.1371/journal.pbio.0060139)
Supplement: Supplementary file 1 [file pbio.0060139.st001.doc]

| ***Inhibitor*** | ***Gametocyte*** | | | ***Ring*** | | | ***Schizont*** | | |
| --- | --- | --- | --- | --- | --- | --- | --- | --- | --- |
| Zaprinast | 33.7 | ± | 1.3 | 3.5 | ± | 1.9 | 3.0 | ± | 1.2 |
| Sildenafil | 75.6 | ± | 1.6 | 19.4 | ± | 1.3 | 22.5 | ± | 1.8 |
| Trequinsin | 192.7 | ± | 2.6 | 26.7 | ± | 1.4 | 126.0 | ± | 1.2 |
| Etazolate | 450.2 | ± | 1.3 | 20.7 | ± | 1.5 | 17.6 | ± | 1.1 |
| Dipyridamole | >200 | | | 6.5 | ± | 1.4 | 7.0 | ± | 1.2 |
| Papaverine | >200 | | | 63.4 | ± | 1.4 | 173.1 | ± | 1.4 |
| Rolipram | >200 | | | >200 | | | >200 | | |
| IBMX | >200 | | | >200 | | | >200 | | |
| Caffeine | >200 | | | n.d. | | | >200 | | |
| Theophylline | >200 | | | n.d. | | | >200 | | |
| **Specific activity of cGMP hydrolysis** | 12.7± 0.5 | | | 17.3± 0.7 | | | 39.5± 0.4 | | |
