# Supplementary material for: Gametogenesis in Malaria Parasites Is Mediated by the cGMP-Dependent Protein Kinase
Source: PLoS Biol. 2008 Jun 3;6(6):e139. doi: 10.1371/journal.pbio.0060139 (PMC2408617; doi:10.1371/journal.pbio.0060139)
Supplement: Supplementary file 2 [file pbio.0060139.st002.doc]

|  | T618Q | | | | T618M | | | |
| --- | --- | --- | --- | --- | --- | --- | --- | --- |
| Comp1 | P | RR | CI | CI | P | RR | CI | CI |
| 0 | 0.272 | 1.044 | 0.967 | 1.127 | 0.114 | 1.031 | 0.993 | 1.072 |
| 0.1 | 0.611 | 1.031 | 0.917 | 1.159 | 0.653 | 1.014 | 0.956 | 1.075 |
| 2 | <0.0001 | 1.827 | 1.666 | 2.003 | 0.458 | 1.020 | 0.968 | 1.074 |
| 4 | n.d. | n.d. | n.d. | n.d. | 0.282 | 0.946 | 0.856 | 1.046 |
| 5 | <0.0001 | 2.248 | 1.904 | 2.652 | 0.843 | 1.010 | 0.916 | 1.113 |
| 6 | <0.0001 | 1.974 | 1.748 | 2.230 | 0.951 | 1.002 | 0.943 | 1.064 |
| 8 | <0.0001 | 1.910 | 1.609 | 2.267 | n.d. | n.d. | n.d. | n.d. |
| 10 | <0.0001 | 1.649 | 1.350 | 2.015 | n.d. | n.d. | n.d. | n.d. |
| 20 | <0.0001 | 1.283 | 1.109 | 1.485 | 0.001 | 0.861 | 0.789 | 0.940 |
